# Prevalence of Zoonotic Parasites and Genetic Diversity of *Ancylostoma caninum* in Dogs From Machakos County, Kenya

**DOI:** 10.1155/japr/1863975

**Published:** 2026-09-23

**Authors:** Camille Glazer, Nicholas Bor, Erastus Mulinge, Maurice K. Murungi, Eric M. Fèvre, Zipporah Gitau, Michael Mugo, Dishon M. Muloi, Neil Sargison, Lian F. Thomas

**Affiliations:** ^1^ Institute of Infection, Veterinary and Ecological Science, University of Liverpool, Leahurst Campus, Neston, UK, liv.ac.uk; ^2^ Health Program, International Livestock Research Institute, Nairobi, Kenya, ilri.org; ^3^ Centre for Microbiology Research, Kenya Medical Research Institute, Nairobi, Kenya, kemri.org; ^4^ R(D)SVS, University of Edinburgh, Easter Bush Campus, Midlothian, UK, ed.ac.uk

**Keywords:** canine, genetic diversity, helminths, hookworms, Kenya, prevalence, zoonotic

## Abstract

**Introduction:**

Soil‐transmitted helminths (STHs) remain a major global public health challenge, with dogs recognised as important reservoirs for several zoonotic species. In Kenya, data on the prevalence and genetic diversity of canine gastrointestinal helminths are scarce. This study investigated the prevalence of zoonotic helminths in dogs and characterised the genetic diversity of canine hookworms in Machakos County, Kenya.

**Methods:**

A cross‐sectional household‐based survey was conducted across three sublocations representing low, medium and high human population density. Faecal samples were collected from 104 dog‐owning households and examined using Mini‐FLOTAC and zinc chloride sieving–flotation techniques. Hookworm species were identified using nested polymerase chain reaction targeting the mitochondrial cytochrome c oxidase subunit I gene, followed by DNA sequencing. Phylogenetic diversity of *Ancylostoma caninum* was assessed using median‐joining haplotype network analyses.

**Results:**

Mini‐FLOTAC detected a higher prevalence of hookworm (89.4% vs. 81.6%), *Toxocara* spp. (45.2% vs. 19.7%) and taeniids (29.8% vs. 5.3%) compared to zinc chloride sieving–flotation. The latter identified additional parasites, including *Trichuris* spp. (9.2%), *Spirocerca lupi* (3.9%), *Alaria* spp. (1.3%) and *Clonorchis sinensis* (2.6%). *A. caninum* was the sole hookworm species identified, alongside genetically distinct *A. caninum*‐like lineages. High genetic variability was observed, comprising 80 polymorphic sites and 40 haplotypes, of which 31 were singletons, seven occurred in two sequences each and two were represented by five and six sequences. Haplotype network and phylogenetic analyses revealed high genetic diversity and weak population structuring in *A. caninum*, with Kenyan isolates clustering with global lineages while exhibiting local diversification. We also report the first molecular confirmation of *Angiostrongylus vasorum* in domestic dogs in Kenya.

**Conclusions:**

These findings underscore dogs as key reservoirs of zoonotic helminths and highlight the need for strengthened surveillance and integrated One Health strategies incorporating molecular diagnostics and targeted education within dog health programmes in line with the WHO NTD Roadmap.

**Impacts**

• Dogs in Machakos County, Kenya, carry a very high burden of intestinal helminths, including several with zoonotic capacity.
• The study provides the first molecular confirmation of the lungworm *Angiostrongylus vasorum* in domestic dogs in Kenya, demonstrating that advanced genetic testing can reveal parasites that routine microscopy misses.
• We highlight dogs as an important but often overlooked source of parasitic infections in communities where humans and animals live in close contact and that an integrated ‘One Health’ approach, which combines dog deworming, public education and improved hygiene, is needed to reduce the risk of zoonotic parasite transmission.

## 1. Introduction

Globally, an estimated 1.5 billion people are infected with soil‐transmitted helminths (STHs), including approximately 10 million Kenyans [1, 2]. While mortality associated with helminth infections is uncommon, their impact on human health is substantial. According to the Global Burden of Disease 2021 estimates, STH infections account for more than 1.38 million disability‐adjusted life years (DALYs) annually, largely due to their adverse effects on nutrition, growth, physical fitness and cognitive development, particularly amongst school‐aged children [3].

Helminth eggs, larvae or segments are shed in the faeces of infected hosts, contaminating the environment until ingested by new hosts, where they colonise the gastrointestinal tract [4]. Many STHs are zoonotic, and dog ownership has been identified as a significant risk factor for human infection, especially in endemic regions such as Kenya [5]. Thus, canine gastrointestinal helminths pose a considerable public health concern due to their potential for zoonotic transmission [6, 7]. In recognition of this threat, the World Health Organization′s Roadmap for Neglected Tropical Diseases 2021–2030 designates several canine‐associated zoonotic helminths as priority targets for elimination by 2030 [8].

There are four primary hookworm species infecting dogs: *Ancylostoma caninum*, *Ancylostoma braziliense*, *Ancylostoma ceylanicum* and *Uncinaria stenocephala*. Of these, *A. caninum* is the most pathogenic, causing fatal haemorrhagic enteritis in puppies and chronic anaemia in older dogs [9]. All four species are zoonotic and can produce cutaneous larva migrans in humans, with *A. braziliense* responsible for severe creeping eruptions and *A. ceylanicum* uniquely capable of establishing patent human infections [10, 11]. Notably, *A. ceylanicum* has emerged as an important zoonosis, increasingly detected in the Asia‐Pacific region, whereas *A. caninum* may occasionally cause eosinophilic enteritis in humans [9]. Similarly, *Toxocara canis* causes visceral and ocular larva migrans, particularly in children [12]. Dogs also act as definitive hosts for *Echinococcus granulosus* sensu lato [13] and several zoonotic *Taenia* species, including *Taenia multiceps* and *Taenia serialis* [14], thereby serving as important reservoirs that sustain transmission cycles and pose significant risks to both livestock and human populations.

Transmission of canine intestinal helminths to humans occurs primarily through contact with contaminated dog faeces, as infective eggs and larvae can persist in the environment for weeks to months under suitable conditions [15]. Environmental contamination not only poses a direct risk to humans but also to livestock, thereby perpetuating a secondary cycle of infection when dogs consume raw or undercooked infected meat or carcasses [16]. Effective control of intestinal helminths in dogs requires improvements in dog husbandry practices alongside the integration of prudent deworming according to parasitological status within public health–focused campaigns, such as rabies vaccination, as well as improved access to companion animal veterinary service provision to enable routine veterinary care for dog populations in LMICs [17]. Public education is essential, focusing on responsible pet ownership (e.g., restricting free roaming), deworming, proper disposal of dog faeces and adoption of basic hygiene practices such as using protective footwear in public spaces. These interventions are consistent with the Tropical Council for Companion Animal Parasite (TroCCAP) guidelines for the diagnosis, treatment and control of canine endoparasites (www.troccap.com/).

Despite the recognised global burden of zoonotic helminths and the established role of dogs as key reservoirs for human infection [6, 7], data on their prevalence and molecular characterisation necessary to determine their potential contribution to human infection in Kenya remain limited [18]. To date, no prevalence data are available for canine populations in Machakos County, limiting understanding of the local epidemiology of zoonotic gastrointestinal helminths and their potential public health impact. Moreover, information on the genetic diversity of *A. caninum* remains limited, as most molecular studies have focused on *A. ceylanicum* because of its established zoonotic importance [19, 20]. This study provides the first baseline data on the prevalence of zoonotic gastrointestinal helminths in dogs from Machakos County and characterises the genetic diversity of *A. caninum*.

## 2. Materials and Methods

This study was embedded in a larger study described in detail in Murungi et al. [21] and summarised briefly here.

### 2.1. Study Site

This study was conducted in Machakos County, Kenya, which has 242 sublocations (third‐level administrative units). Using the human density index as a proxy for urbanisation, three sublocations at the 10th (low), 50th (medium) and 90th (high) percentiles of population density were purposively selected.

### 2.2. Sample Size

The limited available studies on intestinal parasites of dogs in Kenya show considerable variation in zoonotic helminth prevalence across different study sites [18, 22]. Using an assumed average prevalence of 23% in dogs and a precision of 10% at the 95% confidence level, the minimum required sample size was calculated to be 69. This number was then adjusted based on the estimated number of dog‐owning households in the selected sublocations. The final sample size was set at 112 households, to be recruited proportionally from sublocations representing the 10th, 50th and 90th percentiles of human population density.

### 2.3. Household Selection

To select households in each sublocation, global positioning system (GPS) points were randomly generated using ArcGIS software (ArcMap 10.8.1, ESRI). These points were overlaid on satellite imagery with the MAPinR app (Xylem Technologies) to ensure that at least one household structure was located within a 300‐m radius. Points falling within the radius without a household were dropped, and additional random points were generated until the required number of eligible points was achieved. Each point was then visited, and the nearest household within 300 m, starting to the north and proceeding clockwise, was selected for sampling. If residents were unavailable at the initial visit, the household was revisited the following day. Where visible structures on the satellite image turned out not to be households (e.g., shops, schools or churches), these points were excluded and replaced with new randomly generated points to meet the minimum household recruitment target for each sublocation.

### 2.4. Data Collection

After obtaining informed consent, a structured questionnaire was administered to randomly selected households. In households that reported dog ownership, the questionnaire included items on proposed risk factors for helminth infection, such as dog demographics, history of anthelmintic treatment, faecal disposal practices, livestock ownership and deworming and carcass disposal practices. Canine faecal samples were collected from all dog‐owning households. Environmental samples were obtained first, and in households with multiple dogs, as many canine faecal piles as possible were collected into a single pooled sample. Where no faeces could be located in the household environment, sample pots, gloves and verbal instructions on hygienic collection were provided to the primary caregiver. These households were revisited the following day to retrieve the samples. If no environmental samples could be obtained, the study team, comprising qualified veterinarians, attempted a single digital rectal sampling of each dog present at the homestead. Samples were pooled if multiple dogs were present. Rectal sampling was performed only if the participant was comfortable restraining their dog, the dog showed no signs of aggression and only one attempt was made per dog. As part of the study, all participating households received a broad‐spectrum anthelmintic containing praziquantel, pyrantel and fenbendazole. In addition, households were provided with an information leaflet on zoonotic helminth transmission and control (available in English and Kiswahili).

### 2.5. Mini‐FLOTAC Method

All faecal samples were stored in a cool box during transport to the International Livestock Research Institute (ILRI) field laboratory at the Kapiti Research Station. At the laboratory, pooled household faecal samples were weighed, and 2 g aliquots were homogenised in zinc sulphate flotation solution (specific gravity 1.35, verified using a refractometer). The suspensions were then filtered using fill‐FLOTAC devices and transferred into the chambers of a Mini‐FLOTAC device. After a 20‐min settling period, eggs were counted under a light microscope, and counts were multiplied by the dilution factor to calculate the number of eggs per gramme of faeces, stratified by helminth species. Faecal parasitology results were validated via photography shared with coauthors. The remaining faecal material was preserved in absolute ethanol at room temperature and subsequently transported to the Kenya Medical Research Institute (KEMRI) for molecular characterisation.

### 2.6. Zinc Chloride Sieving–Flotation Technique

The zinc chloride sieving–flotation technique (specific gravity 1.45 g/mL) was undertaken at KEMRI to isolate intestinal helminth eggs in the stored faecal samples [23]. Preserved samples were drained of 70% ethanol to obtain faecal pellets. Approximately 2–3 g of homogenised faecal material was transferred into a 15 mL Falcon tube, rinsed with four volumes (8–12 mL) of distilled water and centrifuged at 1600 rpm for 10 min. The supernatant was discarded, and the pellets were washed with four volumes of 1X PBS containing 0.03% Tween‐20, vortexed and centrifuged again at 1600 rpm for 10 min. The resulting pellet was resuspended in four volumes of zinc chloride solution, vortexed and centrifuged at 400 rpm for 30 min to float helminth eggs. The suspension was then passed through 50 and 22 *μ*m sieves (Franz Eckert GmbH, Germany). Eggs retained on the 22 *μ*m sieve were washed into a 15‐mL Falcon tube and centrifuged at 400 rpm for 10 min, and the pellet was transferred into a 2‐mL microcentrifuge tube and stored at 4°C. Concentrated samples were examined microscopically at ×10 objective to identify helminth eggs. Using a micropipette under ×4 magnification, up to 20 individual taeniid and hookworm eggs were picked per sample for further analysis.

### 2.7. DNA Extraction

DNA was extracted from individual hookworm and taeniid eggs or larvae to detect potential mixed infections. Single eggs or larvae were placed in 0.2 mL PCR tubes containing 10 *μ*L of 0.02 M NaOH and lysed at 99°C for 10 min [24]. In parallel, genomic DNA was extracted directly from faecal samples using the QIAamp Fast DNA Stool Mini Kit (Qiagen, Hilden, Germany), following the manufacturer′s protocol with minor modifications. Specifically, eggs were subjected to five freeze‐thaw cycles (−80°C for 30 min followed by 90°C for 15 min) to enhance lysis. DNA was eluted in 50 *μ*L of elution buffer and stored at −20°C until PCR analysis. Genomic DNA from taeniids and hookworms was included as positive controls in the PCR reactions.

### 2.8. Genotyping of Hookworm and Taeniid Eggs

A nested PCR assay targeting the NADH dehydrogenase subunit 1 (nad1) gene was used to detect taeniid eggs, amplifying a ~200 bp fragment as described by Hüttner et al. [25]. For hookworms, a nested PCR targeting the mitochondrial cytochrome oxidase I (CO1) gene was used, producing a 606 bp fragment following the protocol of Mulinge et al. [18]. Each 25 *μ*L PCR reaction contained 2 *μ*L of lysate or DNA template, 1x DreamTaq Green Buffer (20 mM Tris‐HCl [pH 8.0], 1 mM DTT, 0.1 mM EDTA, 100 mM KCl, 0.5% [*v*/*v*] Nonidet P40, 0.5% [*v*/*v*] Tween 20) (Thermo Fisher Scientific, Waltham, MA, United States), 0.2 mM dNTPs (New England Biolabs, Ipswich, MA, United States), 0.25 *μ*M of each forward and reverse primer, 2 mM MgCl_2_ and 0.625 units of DreamTaq Green DNA Polymerase (Thermo Fisher Scientific, Waltham, MA, United States). Amplification conditions were as follows: initial denaturation at 94°C for 5 min; 40 cycles of 94°C for 30 s, 55°C for 30 s and 72°C for 1 min; followed by a final extension at 72°C for 5 min. For taeniids, nadnest A (5 ^′^‐TGT TTT TGA GAT CAG TTC GGT GTG‐3 ^′^) and nadnest C (5 ^′^‐CAT AAT CAA ACG GAG TAC GAT TAG‐3 ^′^) were used in the primary PCR, while nadnest B (5 ^′^‐CAG TTC GGT GTG CTT TTG GGT CTG‐3 ^′^) and nadnest D (5 ^′^‐GAG TAC GAT TAG TCT CAC ACA GCA‐3 ^′^) were used in the secondary PCR [25]. For hookworms, HKF1 and HKR1 were used in the primary PCR and HKF2 and HKR2 in the secondary PCR [18]. PCR products (10 *μ*L) were resolved on 2% agarose gels stained with SYBR Safe DNA Gel Stain (Thermo Fisher Scientific, Waltham, MA, United States) and visualised under a UV transilluminator.

#### 2.8.1. Purification and Sequencing of PCR Amplicons

PCR products were purified using the PureLink Quick PCR Purification Kit (Thermo Fisher Scientific, Waltham, MA, United States) according to the manufacturer′s instructions. Amplicons were eluted in 30 *μ*L of elution buffer and stored at −20°C until further use. The purified products were quantified using a NanoDrop spectrophotometer and sequenced at the Segolip Unit, ILRI, Kenya.

### 2.9. Data Management and Analysis

Parasitology results were first entered into Microsoft Excel, exported as CSV files and subsequently imported into R (R Core Team, 2023; https://www.r-project.org/).

#### 2.9.1. Haplotype Network and Phylogenetic Analysis

DNA sequence data were viewed, edited and assembled using Gentle v.1.9.4 (Manske, [26]; University of Cologne, Germany). The obtained sequences were queried against the National Center for Biotechnology Information (NCBI) nucleotide database using the Basic Local Alignment Search Tool (BLAST) for species identification [27]. Multiple sequence alignment was carried out using the MAFFT online platform (http://mafft.cbrc.jp/alignment/server). A median‐joining haplotype network was constructed in PopART v1.7.63 to visualise relationships amongst haplotypes. Genetic diversity indices, including haplotype diversity (*H* *d*), nucleotide diversity (*π*) and neutrality tests (Tajima′s *D* and Fu′s *F* *s*) [28, 29], were calculated using DnaSP v6.12.03. Phylogenetic relationships were inferred using the maximum likelihood (ML) method based on the Tamura 3‐parameter model, with the robustness of clusters evaluated through 1000 bootstrap replicates. The *Necator americanus cox1* sequence (NC_003416) was used as the outgroup in the phylogenetic analysis.

## 3. Results

A total of 221 households were visited across the three sublocations, and of these, 155 households owned dogs (70%), with an average number of dogs per dog‐owning household of 2.1. Faecal samples were obtained from 107 of the dog‐owning households, with samples not obtained from the remaining 48 as the owners either declined or were unable to collect a sample, and the dog was too aggressive for rectal sampling.

Anthelmintic treatment of dogs was reported by 61 households (57.0%), although 44% of these households could not name the treatment used. Of the households that reported not deworming their dogs, two (4.3%) reported they had no access to treatments, three (6.5%) said dewormers were too expensive and 31 (67.4%) said they believed dewormers were unnecessary for their dogs. The majority of respondents (94%) were aware that dogs can be infected with intestinal helminths, but 57.7% were not aware that canine helminths can infect humans.

### 3.1. Prevalence of Intestinal Helminths as Determined by the Mini‐FLOTAC Method

Sufficient sample material was available for analysis from 104 households. Mixed helminth infections were common, occurring in 90.4% (94/104) of samples. Hookworms were the most prevalent parasites, detected in 89.4% (93/104) of samples, followed by *Toxocara* spp. (45.2%; 47/104), taeniids (29.8%; 31/104) and coccidia (2.9%; 3/104).

### 3.2. Prevalence of Intestinal Helminths as Determined by the Zinc Chloride Sieving–Flotation Method

Seventy‐six preserved samples were sufficient for zinc chloride sieving–flotation and subsequent DNA extraction, and eight species of intestinal parasites were identified. The most prevalent were hookworms (62/76; 81.6%), followed by *Toxocara* spp. (15/76; 19.7%), *Trichuris* spp. (7/76; 9.2%), coccidia (7/76; 9.2%), taeniids (4/76; 5.3%), *Spirocerca lupi* (3/76; 3.9%), *Clonorchis sinensis* (2/76; 2.6%) and *Alaria* spp. (1/76; 1.3%). In addition, seven faecal samples contained unidentified larvae. Results from both tests can be found in Table 1.

**Table 1:** Prevalence of canine intestinal parasites detected using Mini‐FLOTAC and zinc chloride sieving–flotation techniques in Machakos County, Kenya.

Intestinal parasites	Mini‐FLOTAC (*n* = 104) (prevalence %)	Zinc chloride sieving–flotation (*n* = 76) (prevalence %)
Hookworm	93 (89.4)	62 (81.6)
*Toxocara* spp.	47 (45.2)	15 (19.7)
*Trichuris* spp.	—	7 (9.2)
Taeniids	31 (29.8)	4 (5.3)
Coccidian	3 (2.9)	7 (9.2)
*Spirocerca lupi*	—	3 (3.9)
*Alaria* spp.	—	1 (1.3)
*Clonorchis sinensis*	—	2 (2.6)
Larvae^a^		7

^a^Larvae excluded from prevalence estimates as species could not be morphologically identified.

### 3.3. Genotyping of Canine Hookworm and Taeniids

A total of 76 DNA samples extracted directly from faeces were subjected to nested PCR to detect canine hookworms and taeniids. In addition, 506 individual eggs/larvae, including 463 hookworms, 43 taeniids and four unidentified larvae, were lysed and analysed by nested PCR. Of the DNA samples extracted directly from faeces, 12/76 (15.8%) tested positive for hookworm. Amongst the individual eggs, 60/463 hookworm eggs and 1/43 taeniid eggs amplified successfully, while one of the four unidentified larvae was PCR‐positive.

Sequencing of PCR products from both individual eggs and faecal DNA revealed single infections with *A. caninum*. Thirty‐five sequences, 24 derived from eggs and 11 from faecal DNA, showed 97.8%–100% identity with *A. caninum* reference sequences (GenBank Accession Numbers AP017673 and NC_012309) and were therefore confirmed as *A. caninum*. Fourteen additional sequences (13 from eggs and one from faecal DNA) shared 91%–96.7% identity with *A. caninum* and were designated *A. caninum*‐like. One morphologically misidentified hookworm egg was identified as *Oesophagostomum aculeatum*, while the amplicon from one larva corresponded to *A. vasorum*. The nad1 sequence from the single taeniid egg was of insufficient quality to permit species‐level identification. Representative *A. caninum* and *A. caninum*‐like sequences from this study were deposited in GenBank under Accession Numbers OR827067–OR827091 and PP085128–PP085141, respectively (Table 2).

**Table 2:** Sequences of *Ancylostoma caninum* and *A. caninum*‐like from dog faecal samples in Machakos County, Kenya, with matching GenBank references and haplotypes.

Isolate	Species	Accession number	Haplotype	Identity sequence accession number	Identity (%)	Length (bp)	Country
AL436_7	*Ancylostoma caninum*	OR827067	Hap 4	AP017673	99.1	571	Japan
AL437	*Ancylostoma caninum*	OR827068	Hap 2	AP017673	100	583	Japan
AL439	*Ancylostoma caninum*	—	Hap 8	AP017673	99.8	560	Japan
AL442_9	*Ancylostoma caninum*	OR827069	Hap 18	AP017673	99.4	523	Japan
AL445_16	*Ancylostoma caninum*	OR827070	Hap 19	AP017673	99.6	520	Japan
AL448_1	*Ancylostoma caninum*	OR827071	Hap 6	AP017673	99.8	567	Japan
AL448_11	*Ancylostoma caninum*	—	Hap 2	AP017673	100	485	Japan
AL449	*Ancylostoma caninum*	—	Hap 3	AP017673	99.8	561	Japan
AL451	*Ancylostoma caninum*	OR827072	Hap 10	AP017673	99.8	561	Japan
AL452	*Ancylostoma caninum*	OR827073	Hap 12	AP017673	98.9	560	Japan
AL465	*Ancylostoma caninum*	OR827074	Hap 9	AP017673	99.1	563	Japan
AL469_14	*Ancylostoma caninum*	OR827075		AP017673	97.8	276	Japan
AL485_14	*Ancylostoma caninum*	OR827076	Hap 8	AP017673	99.8	566	Japan
AL497	*Ancylostoma caninum*	—	Hap 2	AP017673	100	559	Japan
AL497_6	*Ancylostoma caninum*	OR827077	Hap 3	AP017673	99.8	581	Japan
AL498	*Ancylostoma caninum*	OR827078	Hap 13	AP017673	99.5	560	Japan
AL504_2	*Ancylostoma caninum*	OR827079	Hap 20	AP017673	99.3	543	Japan
AL504_10	*Ancylostoma caninum*	OR827080	Hap 20	AP017673	99.2	514	Japan
AL504_13	*Ancylostoma caninum*	OR827081	Hap 16	AP017673	99.1	542	Japan
AL504_8	*Ancylostoma caninum*	—	Hap 14	AP017673	99.8	491	Japan
AL507	*Ancylostoma caninum*	OR827082	Hap 14	AP017673	99.8	545	Japan
AL507_11	*Ancylostoma caninum*	—	Hap 3	AP017673	99.8	472	Japan
AL507_15	*Ancylostoma caninum*	OR827083	Hap 17	AP017673	99.6	541	Japan
AL508	*Ancylostoma caninum*	OR827084	Hap 5	NC012309	99.7	569	Australia
AL509	*Ancylostoma caninum*	OR827085	Hap 11	AP017673	99.8	561	Japan
AL509_1	*Ancylostoma caninum*	—	Hap 3	AP017673	99.8	555	Japan
AL509_3	*Ancylostoma caninum*	—	Hap 2	AP017673	100	562	Japan
AL509_6	*Ancylostoma caninum*	OR827086	Hap 21	NC012309	99.8	532	Japan
AL509_7	*Ancylostoma caninum*	—	Hap 3	AP017673	99.8	562	Japan
AL509_9	*Ancylostoma caninum*	OR827087	Hap 13	AP017673	99.6	562	Japan
AL509_11	*Ancylostoma caninum*	OR827088	Hap 7	NC012309	98.8	567	Australia
AL509_14	*Ancylostoma caninum*	—	Hap 17	AP017673	99.6	497	Japan
AL509_16	*Ancylostoma caninum*	—	Hap 10	AP017673	99.8	498	Japan
AL509_17	*Ancylostoma caninum*	—	Hap 3	AP017673	99.8	578	Japan
AL509_18	*Ancylostoma caninum*	OR827089	Hap 15	AP017673	99.5	543	Japan
AL497_7	*Angiostrongylus vasorum*	OR827090		NC018602	100	300	Australia
AL508_14	*Oesophagostomum aculeatum*	OR827091		LC428818	98.7	224	Malaysia
AL436_2	*Ancylostoma caninum*‐like	PP085128	Hap 22	AP017673	91.1	529	Japan
AL442_3	*Ancylostoma caninum*‐like	PP085129	Hap 24	OR342776	96.2	498	Thailand
AL442_4	*Ancylostoma caninum*‐like	PP085130		OR342769	95.9	440	Thailand
AL442_18	*Ancylostoma caninum*‐like	PP085131	Hap 23	OR342769	97	572	Thailand
AL445_2	*Ancylostoma caninum*‐like	PP085132	Hap 25	AP017673	93.6	498	Japan
AL445_7	*Ancylostoma caninum*‐like	PP085133	Hap 26	AP017673	94.3	545	Japan
AL457_13	*Ancylostoma caninum*‐like	PP085134	Hap 27	OR342769	96.1	533	Thailand
AL459	*Ancylostoma caninum*‐like	PP085135	Hap 28	OR342769	98.2	558	Thailand
AL469_9	*Ancylostoma caninum*‐like	PP085136	Hap 29	AP017673	95.7	484	Japan
AL490_1	*Ancylostoma caninum*‐like	PP085137	Hap 30	AP017673	95.8	543	Japan
AL490_8	*Ancylostoma caninum*‐like	PP085138	Hap 32	AP017673	96.7	551	Japan
AL495_14	*Ancylostoma caninum*‐like	PP085139	Hap 31	OR342769	96.8	498	Thailand
AL504_9	*Ancylostoma caninum*‐like	PP085140	—	OR342769	97.5	432	Thailand
AL509_20	*Ancylostoma caninum*‐like	PP085141	Hap 33	OR342769	97.6	513	Thailand

### 3.4. Haplotype Network and Phylogenetics

For both haplotype network construction and phylogenetic analysis, all sequences were uniformly trimmed to 463 bp. Sequences OR827075, PP085130 and PP085140 were excluded from further analyses due to their shorter lengths (< 463 bp; Table 2). A total of 56 sequences were retained for haplotype network analysis, comprising 34 *A. caninum* sequences, 12 *A. caninum*‐like sequences generated in this study, three reference sequences (NC012309, FJ483518 and AP017673) and seven additional *A. caninum* sequences from Thailand that matched the trimmed sequence length.

**Figure 1:**
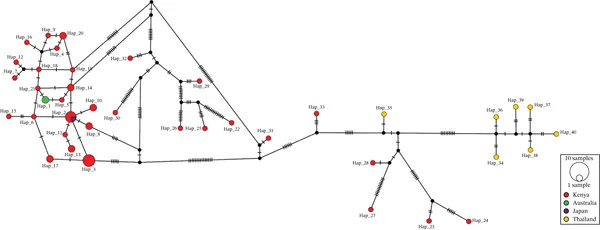
Median‐joining haplotype network based on partial mitochondrial cytochrome c oxidase subunit I gene sequences (463 bp) of *Ancylostoma caninum* isolates obtained from dogs in Machakos County, Kenya.

Analysis of the 463 bp fragment identified 80 polymorphic sites, of which 65 were parsimony‐informative, resulting in the detection of 40 distinct haplotypes. Of the haplotypes identified, 31 were singletons, seven were represented by two sequences each and two by five and six sequences, respectively (Figure 1). The haplotype network revealed a structured pattern of genetic variation centred on a compact Kenyan‐dominated *A. caninum* cluster comprising Haplotypes 1–21, with Hap 2 and Hap 14 as central nodes from which most haplotypes radiated via few mutational steps. Hap 3 was the most frequent haplotype (*n* = 6) and differed from Hap 2 by one mutation; the reference AP017673 clustered within Hap 2, whereas NC012309 and FJ483518 grouped in Hap 1, separated from Hap 2 and Hap 3 by three and four mutations, respectively (Figure 1). Hap 25, Hap 26, Hap 29 and Hap 30 occupied peripheral positions around this core cluster, while Hap 31 and Hap 32 extended along intermediate branches towards more divergent lineages and Hap 22 occurred as a distal terminal branch at the cluster margin. A clearly divergent Kenyan subcluster (Hap 23, Hap 24, Hap 27 and Hap 28) was separated from the main *A. caninum* group by 22–23 mutational steps and connected via the intermediate Hap 31–Hap 33 pathway. This pathway further linked to a highly distinct Thailand‐associated cluster (Hap 34–Hap 40), separated from the Kenyan *A. caninum* haplotypes by 30–32 mutations (Figure 1). Overall haplotype diversity was high (*H* *d* = 0.9792), accompanied by a relatively high nucleotide diversity (*π* = 0.04095). Tajima′s *D* (–0.5989) and Fu′s *F* *s* (–9.533) were both negative but not statistically significant, indicating no strong evidence for recent population expansion.

**Figure 2:**
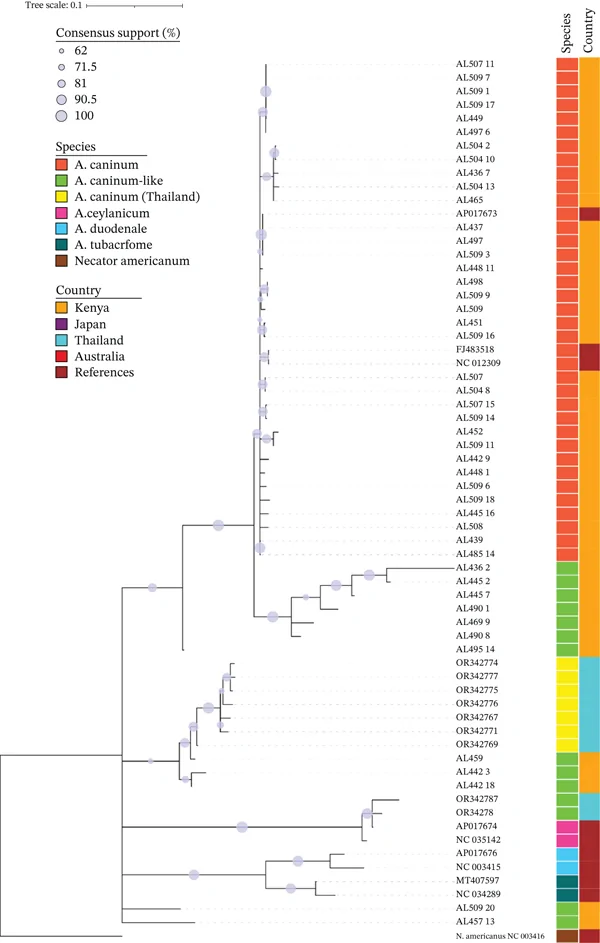
Maximum likelihood phylogenetic tree inferred under the Tamura 3‐parameter model from partial mitochondrial *cox1* sequences (463 bp) of *Ancylostoma caninum* isolates from dogs in Machakos County, Kenya. Branch lengths are scaled to nucleotide substitutions per site. Node support (bootstrap ≥ 60%) is indicated by proportional circles (62%–100%). Tip colour strips denote species identity (*A. caninum*, *A. caninum*‐like, Thailand lineage, *A. ceylanicum*, *A. duodenale*, *A. tubaeforme* and *Necator americanus*) and country of origin (Kenya, Japan, Thailand, Australia and references). *Necator americanus* was used as the outgroup.

The phylogenetic analysis resolved three well‐supported major clades (Figure 2). Clade I comprised all sequences identified as *A. caninum*, including the three reference sequences, and formed a strongly supported monophyletic group; haplotypes within this clade differed by only one to three mutations (Figure 1). Clade II consisted exclusively of *A. caninum*‐like sequences from this study and formed a distinct lineage separated from Clade I by approximately 12–15 mutational steps, consistent with strong bootstrap support (100%). Clade III included *A. caninum*‐like sequences from this study together with *A. caninum* sequences from Thailand and was separated from Clades I and II by approximately 25–32 and 18–23 mutational steps, respectively. Two sequences from this study (AL457_13 and AL509_20), initially classified as *A. caninum*‐like, were positioned basally in the phylogenetic tree between *A. ceylanicum*, *Ancylostoma tubaeforme*, *Ancylostoma duodenale* and *N. americanus*. The phylogenetic topology showed similarity to the genetic groupings observed in the haplotype network (Figure 1).

## 4. Discussion

This study demonstrates a high burden and considerable diversity of intestinal parasites amongst dogs in Machakos County, with hookworms predominating at a prevalence of 81.6%. Both Mini‐FLOTAC and zinc chloride sieving–flotation methods consistently identified hookworms as the dominant parasite group, followed by other nematodes, cestodes and protozoa. This prevalence is markedly higher than that reported from other regions of Kenya, including Narok and Meru counties [18, 22], indicating possible environmental contamination and sustained transmission within the study area. The detection of diverse parasite taxa in dogs underscores the complexity of canine intestinal parasitism and the close health interface between dogs and humans, supporting the need for strengthened surveillance and integrated control to benefit both public and canine health.

*A. caninum* was the only hookworm species detected in this study, consistent with previous reports identifying it as the predominant and, in some areas, the sole canine hookworm species in Kenya [18, 22]. The predominance of *A. caninum* may be attributed to biological advantages, including transmammary transmission and tissue‐stage larval arrest, which facilitate persistence during unfavourable environmental conditions and ongoing transmission within canine populations [9]. Given its association with haemorrhagic enteritis in puppies and chronic iron‐deficiency anaemia in adult dogs, as well as with cutaneous larval migrans and eosinophilic enteritis in humans, *A. caninum* represents a parasite of veterinary and public health concern [10, 30, 31]. The detection of *A. caninum* eggs or DNA in human faeces has increased interest in its potential to infect humans and complete its life cycle; however, current evidence indicates that these findings usually reflect incidental passage rather than true patent infection [32–35].

The detection of less commonly reported helminths, including *O. aculeatum* and the first molecularly confirmed case of *A. vasorum* in domestic dogs in Kenya, highlights previously undocumented parasitological risks and the limitations of morphology‐based diagnosis. *A. vasorum*, a cardiopulmonary nematode of domestic and wild canids transmitted via gastropod intermediate hosts, is associated with severe multisystemic disease and, although widely reported in Europe and parts of the Americas and previously in Uganda, has not been molecularly confirmed in Kenyan dogs until now [36–39]. The frequent misidentification of *O. aculeatum* eggs as hookworm eggs further underscores diagnostic challenges [40, 41]. The lower PCR amplification success from faecal DNA compared with individual eggs highlights the value of egg‐based molecular approaches for accurate parasite identification in mixed infections and for improving the detection of low‐intensity or less abundant species [33, 42]. These methods have been successfully applied to differentiate closely related taeniid eggs and to resolve hookworm species in domestic and wild carnivores [32, 43, 44]. Further epidemiological studies are required to clarify the distribution, prevalence and impact of *A. vasorum* in domestic and wild canid populations in Kenya.

Haplotype network and phylogenetic analyses revealed considerable genetic diversity and weak population structuring in *A. caninum*, consistent with patterns previously reported for this species across broad geographic ranges [45]. The high number of polymorphic and parsimony‐informative sites, together with elevated haplotype and nucleotide diversity, indicates substantial genetic variation within the dataset, while nonsignificant neutrality tests provide no statistical support for recent demographic expansion [29]. The network showed common central haplotypes with multiple closely related variants alongside more divergent lineages, reflecting the coexistence of closely related and genetically distinct haplotypes [46, 47]. Kenyan isolates clustered within the broader global *A. caninum* genetic diversity but also included haplotypes observed only in Kenya, indicating the presence of both shared and region‐specific genetic variants. The similarity of patterns observed between haplotype network and phylogenetic analyses further supports limited population structuring alongside substantial genetic diversity in *A. caninum*. Some *A. caninum*‐like isolates showed low sequence identity to reference *A. caninum* (91%–95%). Notably, AL457_13 and AL509_20 were positioned basally between other *Ancylostoma* spp. and the outgroup (*N. americanus*), indicating marked genetic divergence from the main *A. caninum* clade. Their placement outside both Kenyan *A. caninum* and *A. caninum*‐like clades suggests they may represent an independent lineage within the *A. caninum* complex. However, confirmation of a distinct species will require additional mitochondrial and nuclear loci, morphological characterisation and broader geographic sampling.

Dogs play a significant role in the transmission of zoonotic helminths, acting as reservoirs and sources of environmental contamination with infective stages capable of infecting humans [6, 7]. The high prevalence of hookworm infection observed in this study, together with the detection of other zoonotic helminths such as *Toxocara* spp., *Trichuris* spp. and *O. aculeatum*, highlights the likely contribution of dogs to the substantial STH burden in Kenya [1]. Despite this, control strategies remain largely human‐focused, with mass drug administration programmes prioritising school‐aged children and rarely incorporating dogs or other animal reservoirs [16, 48–50]. Although dog‐targeted deworming has reduced *Echinococcus* transmission in some endemic settings, broader helminth control is limited by reinfection pressure, logistical constraints and concerns regarding anthelmintic resistance [16, 51]. In this context, a shift from elimination towards morbidity reduction through targeted education and awareness is a pragmatic alternative. Consistent with the WHO NTD Roadmap, integrating zoonotic helminth education into existing dog health programmes such as mass vaccination campaigns for rabies offers a feasible One Health approach to reducing transmission and associated human morbidity, particularly in resource‐limited settings [8, 52, 53].

## 5. Conclusion

This study demonstrates a high burden and considerable diversity of intestinal helminths amongst dogs in Machakos County, with *A. caninum* identified as the predominant hookworm species. The detection of multiple zoonotic helminths, including *Toxocara* spp., taeniids, *Trichuris* spp., *O. aculeatum* and *A. vasorum* in domestic dogs in Kenya, underscores the role of dogs as important reservoirs of zoonotic parasites. Molecular analyses revealed high genetic diversity and extensive gene flow in *A. caninum*, consistent with sustained transmission and host movement. Despite the substantial contribution of dogs to the STH burden, current control efforts remain largely human‐focused. These findings highlight the need for strengthened surveillance and integrated One Health strategies that combine molecular diagnostics with targeted education and awareness initiatives embedded within existing dog health programmes, in line with the WHO NTD Roadmap, to reduce zoonotic transmission and associated human morbidity.

## Author Contributions

**Camille Glazer:** conceptualisation, funding acquisition, methodology, investigation, analysis, writing – original draft. **Nicholas Bor:** investigation, analysis, writing – original draft. **Erastus Mulinge:** methodology, analysis, writing – original draft, review and editing. **Maurice K. Murungi:** investigation, writing – review and editing. **Eric M. Fèvre:** supervision, conceptualisation, methodology, writing – review and editing. **Zipporah Gitau:** investigation, writing – review and editing. **Michael Mugo:** investigation, writing – review and editing. **Dishon M. Muloi:** conceptualisation, writing – review and editing. **Neil Sargison:** investigation, writing – review and editing. **Lian F. Thomas:** supervision, conceptualisation, methodology, analysis, writing – review and editing.

## Funding

This study was funded by the Wellcome Trust (10.13039/100010269) (203919/Z/16/Z), the Bundesministerium für Wirtschaftliche Zusammenarbeit und Entwicklung (10.13039/501100006456) and the Consortium of International Agricultural Research Centers (10.13039/501100015815).

## Ethics Statement

Ethical clearance to conduct research with human data was obtained from the Institutional Research Ethics Committee (IREC) of the International Livestock Research Institute (Kenya) (Approval Number ILRI‐IREC 2022‐04/1) and the Institutional Animal Care and Use Committee (IACUC) of ILRI, Kenya (ILRI IREC ILRI‐IACUC2022‐08). ILRI IREC is approved by the Kenyan National Commission for Science, Technology and Innovation (NACOSTI) and approved by the Federal‐wide Assurance for the Protection of Human Subjects in the United States. A research permit was obtained from the NACOSTI for the first author (Licence No. NACOSTI/P/22/1658). The study was also approved by the Ethics Research Committee of the University of Liverpool (United Kingdom) (Approval Number 11201). Local administrative authorities and community leaders provided consent before the start of the study. Informed written consent was obtained from all the study participants.

## Conflicts of Interest

The authors declare no conflicts of interest.

## Data Availability

The data that support the findings of this study are openly available in the University of Liverpool DataCat at https://datacat.liverpool.ac.uk/ (Reference Number 3071).
